# A detailed analysis of borderline results in the QuantiFERON-TB Gold-Plus assay incorporating longitudinal follow-up: intermediate-burden setting

**DOI:** 10.1128/spectrum.02675-25

**Published:** 2025-12-18

**Authors:** Eunju Shin, Changhee Ha, Jong Do Seo, Hanah Kim, Mina Hur, Yeo-Min Yun, Hee-Won Moon

**Affiliations:** 1 Department of Laboratory Medicine, Konkuk University School of Medicine, Seoul, Korea; University of Kentucky, Lexington, Kentucky, USA

**Keywords:** QuantiFERON-TB Gold-Plus, latent tuberculosis infection, borderline, follow-up test, reversion, conversion

## Abstract

The QuantiFERON-TB Gold Plus (QFT-Plus; Qiagen, Hilden, Germany) assay is widely used for latent TB infection (LTBI) screening; however, borderline results often show variability during follow-up. We investigated the longitudinal variability of borderline TB1 and TB2 results throughout the follow-up period. A total of 770 individuals with initial borderline results (0.2–0.7 IU/mL) in either the TB1 or TB2 tube were retrospectively collected over a 5-year period. Agreement and correlation between the initial TB1 and TB2 results were analyzed. Trends in reversion and conversion were evaluated separately for each tube and for combined results among individuals with available follow-up tests. Initial TB1 and TB2 borderline results showed weak agreement (Cohen’s κ = 0.441, 95% CI 0.401–0.480) and moderate correlation (Spearman’s ρ = 0.640, 95% CI 0.596–0.680; *P* < 0.001). TB2 exhibited the highest variability during the first follow-up (33.8%, 47/139), which decreased with subsequent testing. Among those with ≥2 follow-ups, 30.2% (13/43) showed result changes. Cases with borderline values in both TB1 and TB2 showed higher rates of reversion and conversion, whereas having a low-negative result (<0.2 IU/mL) in at least one tube was associated with minimal variability. The QFT-Plus assay provides separate TB1 and TB2 values, and integrating these results may improve interpretation. Follow-up testing is particularly warranted when borderline results occur in both tubes. Further studies are needed to define appropriate criteria and optimal retesting intervals.

**IMPORTANCE:**

This is the first study to longitudinally assess borderline QuantiFERON-TB Gold Plus (QFT-Plus) results in an intermediate TB burden setting, highlighting the need for defined criteria and retesting intervals.

## INTRODUCTION

According to the World Health Organization, an estimated 10.8 million people developed tuberculosis (TB) globally in 2023, and approximately 1.25 million people died from the disease in the same year (1). The Republic of Korea had the second-highest incidence rate and the fifth-highest mortality rate of TB among Organization for Economic Co-operation and Development member countries, with an incidence rate of 38 cases per 100,000 population and a mortality rate of three deaths per 100,000 population in 2023 (2). These figures highlight the burden of TB in Korea and the need for early detection. Individuals with latent tuberculosis infection (LTBI) do not exhibit symptoms of the disease; however, they remain at risk of reactivation, with up to 10% progressing to active TB during their lifetime (3). To reduce the incidence of tuberculosis, it is essential to identify individuals with LTBI and provide them with appropriate preventive treatment and management (4).

The QuantiFERON-TB Gold Plus (QFT-Plus; Qiagen, Hilden, Germany), which incorporates an additional TB2 tube into the previous version (QuantiFERON-TB Gold In-Tube, QFT-GIT), has been widely used among interferon-gamma release assays (IGRAs). While the TB1 tube is designed to elicit an interferon-gamma (IFN-γ) response from CD4+ helper T lymphocytes, the TB2 tube includes additional peptides that stimulate responses from both CD4+ helper T cells and CD8+ cytotoxic T lymphocytes, an important component of host immunity against *Mycobacterium tuberculosis* (4–6). QFT-Plus has been reported to demonstrate higher sensitivity than QFT-GIT in detecting active tuberculosis and early infection (4–8).

However, individuals with borderline results frequently exhibit reversion from positive to negative or conversion from negative to positive upon follow-up testing (9–14). These findings highlight the variability of QFT-Plus results within the borderline range, suggesting that reversions or conversions may lead to overtreatment or missed opportunities for appropriate care. In this study, we retrospectively collected individuals with borderline QFT-Plus results and conducted a detailed analysis of the relationship between TB1-Nil and TB2-Nil values. Additionally, we examined their longitudinal variability over the follow-up period and compared trends in follow-up testing outcomes based on the stratification of TB1-Nil and TB2-Nil values.

## MATERIALS AND METHODS

### Study population

This *in vitro* study was conducted based on data collected from June 2019 to February 2025 at Konkuk University Medical Center in Seoul, Korea. During this period, 9,645 QFT-Plus tests were performed. Of these, 770 individuals (8.0%) with initial borderline IFN-γ responses (0.2–0.7 IU/mL) in either the TB1-Nil or TB2-Nil were included.

The study population consisted of 364 men (47.3%) and 406 women (52.7%), with a median age of 58 years (interquartile range [IQR], 44–67.8 years). The department that requested the highest number of QFT-Plus tests was family medicine (249, 32.3%), followed by dermatology (159, 20.6%), pulmonology (130, 16.9%), and rheumatology (87, 11.3%).

Among those tested, 499 (64.8%) underwent LTBI screening due to immunosuppression or planned immunosuppressive therapy, 249 (32.3%) were healthcare workers (HCWs) undergoing routine screening, 7 (0.9%) were tested following known exposure to active TB, and 15 (1.9%) were evaluated for suspected active TB. Of these 15 individuals, 12 were subsequently diagnosed with active TB and initiated treatment; however, none underwent follow-up QFT-Plus testing during or after therapy. In addition, four other individuals diagnosed with active TB during the study period (excluding those initially suspected of having active TB) also did not undergo follow-up QFT-Plus testing after treatment.

**Fig 1:**
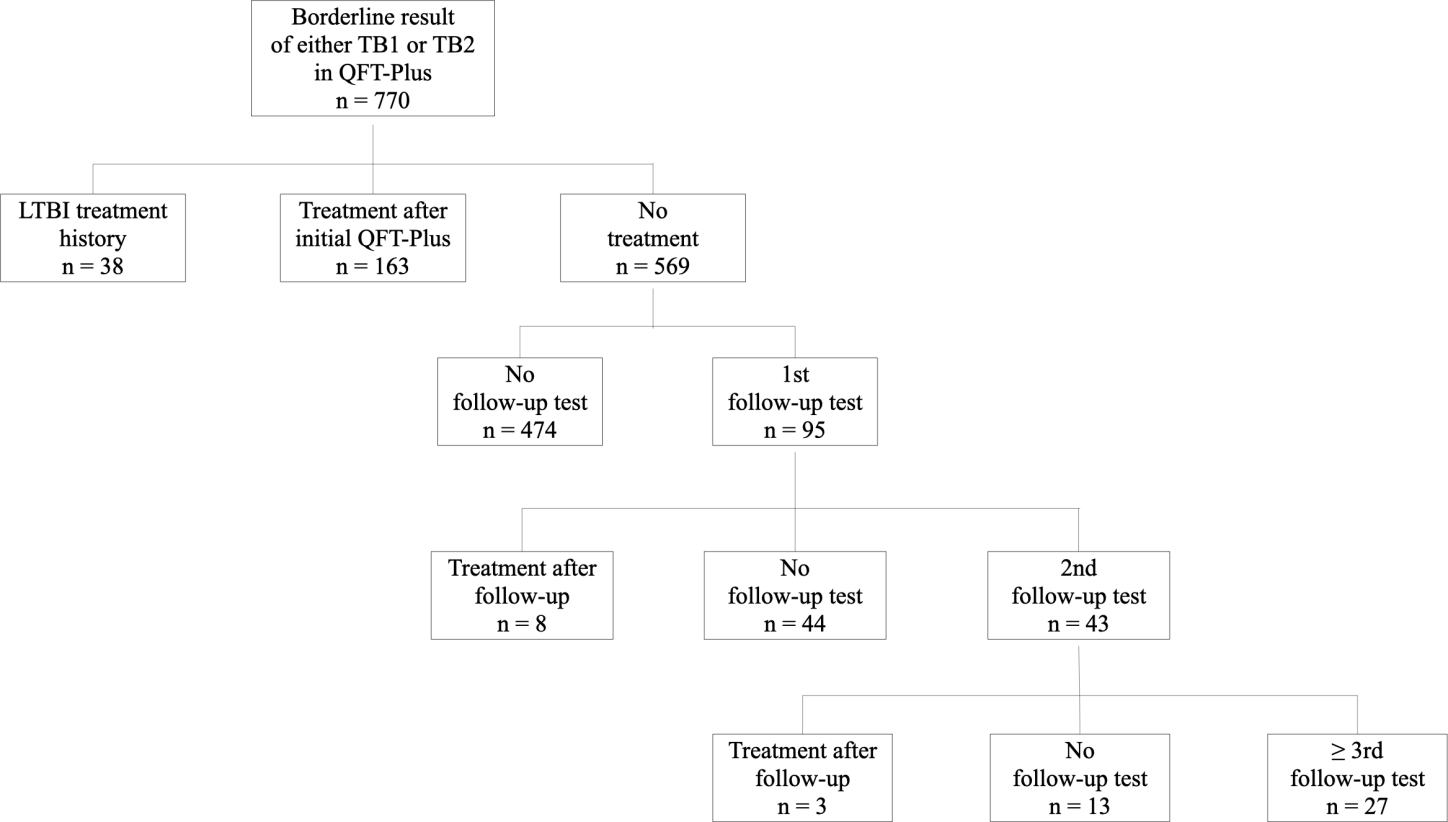
Flowchart showing the distribution of individuals with borderline QuantiFERON-TB Gold Plus results, categorized by LTBI treatment status and follow-up testing. LTBI, latent TB infection.

Among the 770 individuals included in the study, 38 had a history of prior LTBI treatment. A total of 139 individuals underwent a first follow-up QFT-Plus test, of whom 68 received a second follow-up, 34 a third, and 20 a fourth follow-up test. When excluding individuals who had received LTBI treatment, 95 underwent a first follow-up test, 43 underwent a second, and 27 underwent three or more follow-up tests (Fig. 1). This study was conducted using medical records and did not require any study-specific interventions or additional blood sampling. Therefore, the Institutional Review Board of KUMC approved the study protocol (KUMC 2025-04-020) with waived informed consent.

### QuantiFERON-TB Gold-Plus test

All study samples were analyzed using QFT-Plus. The assay uses a specialized blood collection system consisting of four tubes: a negative control (Nil), TB1, TB2, and a positive control (Mitogen). The test was performed according to the manufacturer’s instructions (15). The tubes were incubated at 37°C for 16 to 24 h immediately after collection. Plasma was then separated and stored at 4°C. The concentration of IFN-γ in each tube was measured, and the result was considered positive if either TB1-Nil or TB2-Nil was ≥0.35 IU/mL and at least 25% greater than the Nil value. However, previous studies (9, 11, 12, 14, 16) have reported high variability in follow-up results among individuals with borderline responses, defined as TB1-Nil or TB2-Nil values between 0.2 and 0.7 IU/mL. Therefore, this study defined the borderline range as 0.2–0.7 IU/mL. To allow for a more detailed analysis, we further refined this range by subdividing it into two categories: borderline positive (0.35–0.69 IU/mL) and borderline negative (0.2–0.34 IU/mL), based on a cutoff value of 0.35 IU/mL.

Medical records were reviewed, and subjects were categorized according to the interval between the initial and follow-up QFT-Plus tests: ≤6 months, 7–12 months, and >12 months. Test results were evaluated for conversion and reversion. TB1-Nil and TB2-Nil results were analyzed from the initial test, with borderline results up to the fourth follow-up test.

During the study period, frequencies of reversion and conversion were evaluated in individuals undergoing follow-up testing. Reversion and conversion rates were also analyzed separately for each tube (TB1 and TB2) based on respective values. For individuals with two or more follow-up tests, results were classified as transient or consistent according to observed trends. Follow-up test data obtained after LTBI treatment were excluded from analysis. Additionally, TB1-Nil and TB2-Nil follow-up results before and after LTBI treatment were analyzed separately.

### Statistical analysis

The strength and direction of the linear relationship between initial TB1-Nil and TB2-Nil were assessed using Spearman’s correlation coefficient. The coefficients were interpreted as follows: <0.30, negligible; 0.30–0.50, low; 0.50–0.70, moderate; 0.70–0.90, high; and 0.90–1.00, very high (17). The agreement between initial TB1-Nil and TB2-Nil was calculated using Cohen’s kappa (κ) with 95% CI, which was interpreted as follows: ≤0.20, none; 0.21–0.39, minimal; 0.40–0.59, weak; 0.60–0.79, moderate; 0.80–0.90, strong; and >0.90, nearly perfect (18).

A Bland-Altman plot and Passing-Bablok regression analysis were performed to compare TB1-Nil and TB2-Nil, according to CLSI guideline EP09C-ED3 (19). The absolute mean difference in IFN-γ levels between TB1-Nil and TB2-Nil was analyzed using a Bland-Altman plot. IFN-γ levels between TB1-Nil and TB2-Nil were compared using Passing-Bablok regression.

Statistical analyses were performed using Microsoft Excel Software (version 2019; Microsoft Corporation, Redmond, WA, USA) and MedCalc Statistical Software (version 23.2.6; MedCalc Software, Ostend, Belgium); *P* < 0.05 was considered statistically significant.

## RESULTS

### Quantitative and qualitative comparisons between TB1 and TB2 tubes with borderline range

**Fig 2:**
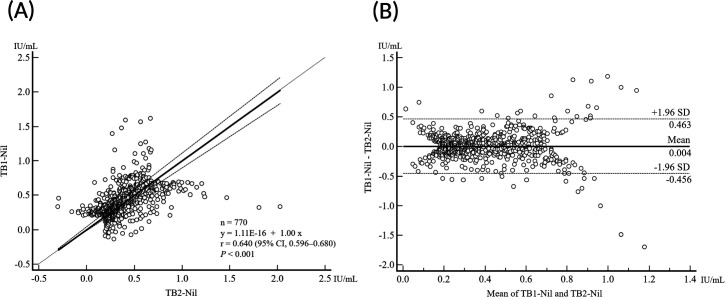
Correlation between TB1-Nil and TB2-Nil values within the borderline range in QuantiFERON-TB Gold-Plus test (*n* = 770). (**A**) Passing Bablok regression. (**B**) Bland-Altman plot.

Figure 2 shows a comparison of initial borderline QFT-Plus test results between TB1 and TB2 tubes, using Passing-Bablok regression analysis and a Bland-Altman plot. A moderate correlation was observed between TB1-Nil and TB2-Nil (r = 0.640; 95% CI, 0.596 to 0.680; *P* < 0.001), with a mean difference of 0.004 IU/mL (95% CI, –0.013 to 0.020) (Fig. 2A and B).

The agreement between TB1 and TB2 tubes was weak (κ = 0.441; 95% CI, 0.401 to 0.480). Among the 770 individuals tested, 59.6% (459/770) had positive results, and 40.4% (311/770) had negative results according to the QFT-Plus interpretation criteria. Using the manufacturer’s cutoff criteria, the concordance between TB1 and TB2 tubes was 79.2% (610/770), with discordance observed in 20.8% (160/770). However, when the borderline range was further subdivided into two categories—borderline positive (0.35–0.69 IU/mL) and borderline negative (0.2–0.34 IU/mL)—the concordance between the TB1 and TB2 tubes was reduced to 39.9% (307/770), while discordance increased to 60.1% (463/770) (Table 1).

**TABLE 1:** Agreement between initial TB1-Nil and TB2-Nil values within the borderline ranges^*a*^

TB2-Nil (IU/mL)	TB1-Nil (IU/mL)					Kappa (95% CI)
	Negative (<0.2)	Borderline negative (0.2–0.34)	Borderline positive (0.35–0.69)	Positive (≥0.7)	Total	
Negative (<0.2)	0	99	15	0	114	0.441 (0.401–0.480)
Borderline negative (0.2–0.34)	86	126	61	7	280	
Borderline positive (0.35–0.69)	19	51	180	58	308	
Positive (≥0.7)	0	7	60	1	68	
Total	105	283	316	66	770	

^*a*^ The gray area indicates values classified as negative according to the QFT-Plus interpretation criteria, whereas all remaining values are interpreted as positive.

### Follow-up test results in TB1 and TB2 tubes according to test intervals

TB1 and TB2 tube results were analyzed from the first to the fourth follow-up tests, stratified by follow-up intervals of <6 months, 7–12 months, and >12 months. At the first follow-up, the highest rates of reversion and conversion were observed in the group with a follow-up interval <6 months (TB1, 38.9%; TB2, 55.5%). Changes in TB1 and TB2 tube results were more pronounced between the first and second follow-up tests. The overall variation rate in the TB1 tube, including both conversions and reversions, was 22.3% (31/139) at the first follow-up, decreasing to 16.2% (11/68) at the second. For the TB2 tube, the variation rate was 33.8% (47/139) at the first follow-up, and 16.2% (11/68) at the second. As the number of follow-up tests increased, variability in TB2 tube results decreased more than in TB1 tube results (Table 2).

**TABLE 2:** Follow-up intervals and QuantiFERON-TB Gold-Plus results in individuals with repeat testing^*a*^

	First follow-up test interval (median = 13 mo, range = 0–59 mo, IQR = 11–16 mo), *N* = 139					
	≤6 mo (*N* = 18)		7–12 mo (*N* = 43)		>12 mo (*N* = 78)	
	TB1 (%)	TB2 (%)	TB1 (%)	TB2 (%)	TB1 (%)	TB2 (%)
Conversion	1 (5.6)	4 (22.2)	5 (11.6)	5 (11.6)	8 (10.3)	10 (12.8)
Reversion	6 (33.3)	6 (33.3)	1 (2.3)	8 (18.6)	10 (12.8)	14 (17.9)

	Second follow-up test interval (median = 13 mo, range = 2–44 mo, IQR = 11–14 mo), *N* = 68					
	≤6 mo (*N* = 4)		7–12 mo (*N* = 28)		>12 mo (*N* = 36)	
	TB1 (%)	TB2 (%)	TB1 (%)	TB2 (%)	TB1 (%)	TB2 (%)
Conversion	0 (0.0)	2 (50.0)	1 (3.6)	1 (3.6)	4 (11.1)	4 (11.1)
Reversion	2 (50.0)	1 (25.0)	0 (0.0)	1 (3.6)	4 (11.1)	2 (5.6)

	Third follow-up test interval (median = 12 mo, range = 8–19 mo, IQR = 11–14 mo), *N* = 34					
	≤6 mo (*N* = 0)		7–12 mo (*N* = 22)		>12 mo (*N* = 12)	
	TB1 (%)	TB2 (%)	TB1 (%)	TB2 (%)	TB1 (%)	TB2 (%)
Conversion	0 (0.0)	0 (0.0)	2 (9.1)	1 (4.5)	0 (0.0)	1 (8.3)
Reversion	0 (0.0)	0 (0.0)	0 (0.0)	0 (0.0)	0 (0.0)	1 (8.3)

	Fourth follow-up test interval (median = 11 mo, range = 8–17 mo, IQR = 10–13 mo), *N* = 20					
	≤6 mo (*N* = 0)		7–12 mo (*N* = 13)		>12 mo (*N* = 7)	
	TB1 (%)	TB2 (%)	TB1 (%)	TB2 (%)	TB1 (%)	TB2 (%)
Conversion	0 (0.0)	0 (0.0)	1 (7.7)	1 (7.7)	1 (14.3)	1 (14.3)
Reversion	0 (0.0)	0 (0.0)	0 (0.0)	0 (0.0)	1 (14.3)	0 (0.0)

^*a*^ LTBI, latent TB infection.

### Follow-up test results in TB1 and TB2 tubes excluding individuals receiving LTBI treatment

Table 3 shows follow-up result changes stratified by initial TB1-Nil and TB2-Nil values. In the TB1 tube, 20.7% (6/29) of individuals with an initial borderline-negative result (0.20–0.34 IU/mL) showed conversion, whereas 55.6% (5/9) of those with an initial borderline-positive result (0.35–0.69 IU/mL) showed reversion. In the TB2 tube, conversion occurred in 28.6% (4/14) of those with borderline-negative results, and reversion was observed in 81.8% (9/11) of those with borderline-positive results. Individuals with low negative results (<0.2 IU/mL) in the TB1 or TB2 tubes showed predominantly consistent negative responses upon follow-up (100% in TB1 and 93.8% in TB2).

**TABLE 3:** Trends in follow-up results stratified by initial quantitative TB1-Nil and TB2-Nil values from the QuantiFERON-TB Gold-Plus assay in individuals with ≥2 follow-up tests, excluding those who received LTBI treatment (*N* = 43)^*a*^

TB1-Nil (IU/mL)	Overall trend						Total (*N* = 43)
	Consistent negative (%)	Consistent positive (%)	Consistent conversion (%)	Transient conversion (%)	Consistent reversion (%)	Transient reversion (%)	
Negative (<0.2)	4 (100.0)	0 (0)	0 (0)	0 (0)	0 (0)	0 (0)	4
Borderline negative (0.2–0.34)	23 (79.3)	0 (0)	3 (10.3)	3 (10.3)	0 (0)	0 (0)	29
Borderline positive (0.35–0.69)	0 (0)	4 (44.4)	0 (0)	0 (0)	3 (33.3)	2 (22.2)	9
Positive (≥0.7)	0 (0)	0 (0)	0 (0)	0 (0)	0 (0)	1 (100.0)	1
TB2-Nil (IU/mL)							
Negative (<0.2)	15 (93.8)	0	0	1 (6.3)	0	0	16
Borderline negative (0.2–0.34)	10 (71.4)	0	1 (7.1)	3 (21.4)	0	0	14
Borderline positive (0.35–0.69)	0	2 (18.2)	0	0	6 (54.5)	3 (27.3)	11
Positive (≥0.7)	0	1 (50.0)	0	0	0	1 (50.0)	2

^*a*^ LTBI, latent TB infection.

Changes in follow-up results based on combined TB1 and TB2 categories are presented in Table 4. When either the TB1 or TB2 tube initially showed a borderline result (0.2–0.7 IU/mL), the overall rate of change—including both reversion and conversion—was 30.2% (13/43). Among individuals with borderline TB2 results with low negative TB1 results (<0.2 IU/mL), 75% (3/4) demonstrated consistent follow-up results. Similarly, when TB1 results were borderline and TB2 results were negative, 93.8% (15/16) showed consistent results. In total, 90% (18/20) of individuals with low negative results in at least one tube showed consistent outcomes. In contrast, among those with borderline results in both TB1 and TB2, reversion or conversion was observed in 45% (9/20) of cases.

**TABLE 4:** Trends in follow-up results based on initial borderline TB1 and TB2 tube values from the QuantiFERON-TB Gold-Plus assay in individuals with ≥2 follow-up tests, excluding those who received LTBI treatment (*N* = 43)^*a*^^,^^*b*^^,^^*c*^

TB1-Nil (IU/mL)	TB2-Nil (IU/mL)	N	Consistent (%)	Reversion (%)	Conversion (%)
Negative (<0.2)	Negative (<0.2)	0	0	0	0
	Borderline negative (0.2–0.34)	3	3 (100.0)	0	0
	Borderline positive (0.35–0.69)	1	0	1 (100.0)	0
	Positive (≥0.7)	0	0	0	0
Borderline negative (0.2–0.34)	Negative (<0.2)	16	15 (93.75)	0	1 (6.25)
	Borderline negative (0.2–0.34)	11	7 (63.6)	0	4 (36.4)
	Borderline positive (0.35–0.69)	2	0	2 (100)	0
	Positive (≥0.7)	0	0	0	0
Borderline positive (0.35–0.69)	Negative (<0.2)	0	0	0	0
	Borderline negative (0.2–0.34)	0	0	0	0
	Borderline positive (0.35–0.69)	7	4 (57.1)	3 (42.9)	0
	Positive (≥0.7)	2	1 (50.0)	1 (50.0)	0
Positive (≥0.7)	Negative (<0.2)	0	0	0	0
	Borderline negative (0.2–0.34)	0	0	0	0
	Borderline positive (0.35–0.69)	1	0	1 (100.0)	0
	Positive (≥0.7)	0	0	0	0

^*a*^ Results after latent TB infection treatment were excluded.

^*b*^ Consistent results include consistent positive and consistent negative. Reversion results include consistent reversion and transient reversion. Conversion results include consistent conversion and transient reversion.

^*c*^ LTBI, latent TB infection.

Among the 770 individuals with initial borderline IFN-γ responses (0.2–0.7 IU/mL) in either TB1-Nil or TB2-Nil, 15 individuals who underwent testing from baseline through the fourth follow-up (excluding those who received LTBI treatment) showed that 46.7% (7/15) experienced either reversion (4/15) or conversion (3/15), while the remaining 53.3% (8/15) had consistently negative results (Table 5). The median coefficients of variation (CVs) for TB1 and TB2 were 61.98% (95% CI, 38.64 to 80.91) and 62.96% (95% CI, 46.63 to 77.18), respectively, indicating substantial variability. No statistically significant difference was found between the CVs of TB1 and TB2 (*P* = 0.934). Furthermore, there was no significant difference in CV values between individuals with variable test results (conversion or reversion) and those with stable results for both TB1 (*P* = 0.779) and TB2 (*P* = 0.189) (Table 5).

**TABLE 5:** Detailed results of QuantiFERON-TB Gold-Plus TB1-Nil and TB2-Nil in individuals who underwent the first test through the fourth follow-up test, excluding those who received LTBI treatment (*N* = 15)^*a*^^,^^*b*^

Case no.	First assessment			Second assessment			Third assessment			Fourth assessment			Fifth assessment			CV (%)		Overall trend
	TB1	TB2	Result	TB1	TB2	Result	TB1	TB2	Result	TB1	TB2	Result	TB1	TB2	Result	TB1	TB2	
1	0.55	0.66	P	0.38	0.32	P	0.17	0.14	N	0.26	0.28	N	0.32	0.37	P	42.44	54.03	Transient reversion
2	0.14	0.35	P	0.03	−0.02	N	0.01	0.04	N	−0.01	0.03	N	−0.03	0.01	N	237.44	184.83	Consistent reversion
3	0.35	0.42	P	0.11	0.1	N	0.18	0.18	N	0	0	N	0.14	0.22	N	81.66	85.03	Consistent reversion
4	0.3	0.69	P	0.06	0.11	N	0.3	0.22	N	0.5	0.29	P	0.17	0.19	N	61.98	75.79	Consistent reversion
5	0.21	0.17	N	0.24	0.13	N	0.3	0.23	N	0.16	0.18	N	0.47	0.39	P	43.37	46.13	Transient conversion
6	0.29	0.27	N	0.05	0.07	N	0.41	0.33	P	0.47	0.5	P	1	0.94	P	78.82	77.68	Consistent conversion
7	0.31	0.27	N	0.41	0.57	P	0.21	0.15	N	0.4	0.29	P	0.4	0.26	P	24.91	50.74	Consistent conversion
8	0.21	0.17	N	0.13	0.1	N	0.13	0.12	N	0.09	0.06	N	0.13	0.11	N	31.75	35.38	Consistent negative
9	0.31	0.14	N	0.32	0.27	N	0.02	0.09	N	0.14	0.25	N	0.17	0.11	N	65.41	48.01	Consistent negative
10	0.23	0.18	N	0.06	0.07	N	0.08	0	N	0.07	0.08	N	0.08	0.12	N	68.19	73.70	Consistent negative
11	0.09	0.22	N	0.03	0.05	N	0.09	0.04	N	0.11	0.08	N	0.1	0.15	N	37.27	70.33	Consistent negative
12	0.31	0.26	N	−0.01	0.01	N	0.01	0.02	N	0.21	0.08	N	0.01	0	N	136.97	146.67	Consistent negative
13	0.3	0.16	N	−0.41	−0.19	N	0.01	0.03	N	−0.07	−0.08	N	0	0	N	−745.98	−813.22	Consistent negative
14	0.29	0.18	N	0.05	0.03	N	0.08	0.08	N	0.05	0.07	N	0.07	0.08	N	94.97	62.96	Consistent negative
15	0.31	0.2	N	0.16	0.08	N	0.09	0.13	N	0.27	0.23	N	0.17	0.2	N	44.44	36.55	Consistent negative

^*a*^ TB1 and TB2 values refer to TB1-Nil and TB2-Nil (IU/mL), respectively.

^*b*^ LTBI, latent TB infection; P, positive; N, negative.

### Follow-up test results in TB1 and TB2 tubes in individuals with LTBI treatment

**Fig 3:**
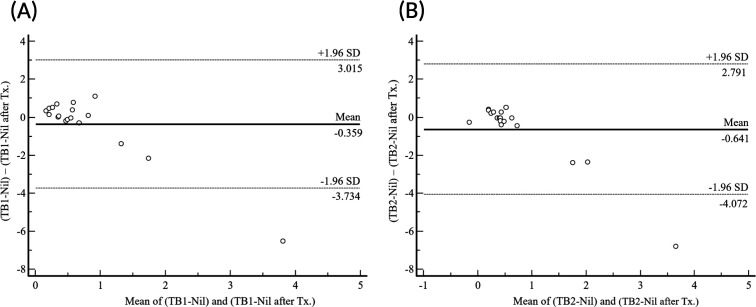
Bland-Altman plots comparing TB1-Nil values before and after LTBI treatment (**A**) and TB2-Nil values before and after LTBI treatment (**B**) (*n* = 18). LTBI, latent TB infection; Tx., treatment.

Among the 770 individuals with borderline results, a total of 174 (22.6%) received treatment: 163 after the initial test, 8 after the second test, and 3 after the third test (Fig. 1). Follow-up results were available for 18 of these individuals after treatment. Bland-Altman analysis was performed to compare TB1 and TB2 values before and after treatment. The absolute mean differences in TB1 and TB2 were –0.359 (95% CI, –1.216 to 0.497) and –0.641 (95% CI, –1.511 to –0.230), respectively (Fig. 3).

In the TB1 tube, 33.3% (6/18) of subjects exhibited reversion, while in the TB2 tube, 55.6% (10/18) demonstrated either reversion or conversion. However, no statistically significant differences were observed in TB1-Nil and TB2-Nil values before and after treatment (*P* = 0.695 and *P* = 0.248, respectively), as detailed in Table 6.

**TABLE 6:** The results of QuantiFERON-TB Gold-Plus TB1-Nil and TB2-Nil results before and after LTBI treatment^*a*^

Subject number	TB1-Nil (IU/mL)					Overall trend	TB2-Nil (IU/mL)					Overall trend
	Before Tx.	Follow-up after Tx.					Before Tx.	Follow-up after Tx.				
		First	Second	Third	Fourth			First	Second	Third	Fourth	
1	0.52	0.02	0.17	0.1	0.15	Reversion	0.42	0.17	0.22	0.17	0.18	Reversion
2	0.67	0				Reversion	0.38	0.03				Reversion
3	0.97	0.21				Reversion	0.79	0.28				Reversion
4	0.39	0.34				Reversion	0.31	0.53				Conversion
5	0.45	−0.01				Reversion	−0.29	−0.01				Negative
6	0.52	0.84	0.28	0.21	0.67	Reversion	0.51	0.97	0.41	0.42	0.4	Positive
7	0.33	0.01				Negative	0.4	0.01				Reversion
8	0.28	0.16				Negative	0.35	0.15				Reversion
9	0.76	0.39				Positive	0.57	0.32				Reversion
10	0.54	7.09				Positive	0.25	7.08				Conversion
11	0.43	0.57				Positive	0.34	0.4				Conversion
12	0.37	0.58				Positive	0.23	0.65				Conversion
13	1.47	0.38	5.95	1.65	1.09	Positive	0.37	0.62	6.56	1.07	1.84	Positive
14	0.62	2.03	0.81			Positive	0.55	2.97	0.97			Positive
15	0.35	0.37				Positive	0.4	0.45				Positive
16	0.86	0.78				Positive	0.61	0.66				Positive
17	0.66	2.83				Positive	0.84	3.23				Positive
18	0.52	0.59				Positive	0.38	0.43				Positive

^*a*^ LTBI, latent TB infection; Tx., treatment.

## DISCUSSION

We investigated the relationship between TB1 and TB2 in individuals with borderline QFT-Plus results and assessed the longitudinal variability of these borderline values throughout the follow-up period. Given that study outcomes may vary depending on the TB prevalence in different populations, further studies in settings with varying TB burdens are warranted. To our knowledge, no follow-up study focusing on borderline QFT-Plus results has been conducted in intermediate TB burden countries. In this study, we specifically evaluated follow-up trends by stratifying TB1 and TB2 values individually, as well as analyzing combined test outcomes. Given the study population’s characteristics—specifically, a relatively high proportion of positive baseline results and a substantial number receiving LTBI treatment—we analyzed treated and untreated individuals separately to distinguish between treatment-related changes and spontaneous variability. In addition, we performed a comparative analysis of QFT-Plus results before and after LTBI treatment.

Although previous studies have demonstrated a high correlation and excellent agreement between TB1 and TB2 values in the QFT-Plus assay (13, 20, 21), our findings revealed only moderate correlation and weak agreement when analysis was limited to samples with borderline results (Fig. 2; Table 1). Although a previous meta-analysis reported significantly higher IFN-γ levels in the TB2 tube compared to TB1 (8), no significant difference between TB1 and TB2 values was observed within the borderline range in our study (*P* = 0.609). Given that the QFT-Plus interpretation algorithm considers a test positive if either the TB1 or TB2 result exceeds the cutoff, the low concordance between the two tubes in the borderline range suggests that a single measurement may be insufficient for definitive diagnosis in this subgroup.

IGRAs show considerable variability, particularly near cutoff values, highlighting the need for follow-up testing to avoid delayed LTBI treatment due to false negatives and unnecessary therapy from false positives (22–27). It has been suggested in one study that QFT-GIT results falling within a borderline zone of 0.2–0.7 IU/mL in HCWs should be retested (23). A recent study from low-TB burden Sweden showed that over one-third of initial borderline QFT-Plus results reverted to negative on retesting, supporting the clinical utility of introducing a borderline range for QFT-Plus interpretation (13). To date, no definitive guidelines exist regarding the timing of QFT-Plus follow-up testing or the optimal retesting interval for individuals with borderline results.

Although our institution recommends follow-up testing for individuals with borderline QFT-Plus results, no specific retesting interval is currently suggested. Among 770 individuals with borderline results in our retrospective analysis, only 2.9% (22/770) underwent repeat testing within 6 months. Notably, most follow-up tests were performed beyond the 6-month window, suggesting that these were conducted as part of routine serial testing rather than targeted reassessment of indeterminate results.

We focused exclusively on subjects with borderline QFT-Plus results and stratified the follow-up intervals into three groups—less than 6 months, 7–12 months, and more than 12 months—to perform a more detailed analysis. In serial testing, variability was greatest within the first year, particularly at the earliest follow-up. TB2 values demonstrated greater fluctuation compared to TB1 (Table 2), consistent with prior reports suggesting that the TB2 tube may contribute to increased variability in IGRA results (25, 27). However, the interpretation of these findings may vary depending on the tested population, such as whether individuals were undergoing routine health screening or had known tuberculosis exposure (7, 13, 22, 23). Accordingly, separate studies are needed to evaluate the clinical significance of borderline results based on patient risk profiles.

Individuals who had borderline results in both TB1 and TB2 at baseline showed higher rates of reversion or conversion during follow-up (Table 4). These findings are consistent with those reported in the Swedish study (13) and support the need for introducing a borderline range in the interpretation of QFT-Plus results, even in intermediate TB burden settings. Furthermore, we observed that variability decreased as the number of follow-up tests increased, suggesting that repeated follow-up testing may not be necessary. The QFT-Plus assay uses two separate antigen tubes, which may introduce some inconvenience due to the need for additional blood collection. However, obtaining two distinct measurements allows for combined interpretation in cases of borderline results, potentially reducing variability. In our study, despite frequent occurrences of reversion and conversion within the borderline zone, follow-up test results were largely consistent when at least one of the two tubes demonstrated a low negative result (<0.2 IU/mL). With larger data sets in the future, it is anticipated that more refined interpretative criteria based on the individual and combined values of TB1 and TB2 tubes can be proposed. Furthermore, analysis of the CV from subjects with multiple serial test results revealed substantial variability in both TB1 and TB2 responses, indicating that QFT-Plus results may fluctuate considerably within the same individual over time (Table 5). This variability could be attributed to unstable immunological responses or intrinsic assay variability (7, 27), underscoring the need for additional clinical evaluation within the appropriate context.

Although the sample size was limited, this study enabled an assessment of QFT-Plus responses before and after LTBI treatment. The mean difference in interferon-gamma responses before and after treatment was negative; however, this did not indicate a statistically significant directional trend. Inter-individual variability was observed, with greater fluctuations in TB2 responses compared to TB1, although this difference did not reach statistical significance (Fig. 3). Longitudinal analysis demonstrated that 33.3% (6/18) of participants exhibited variability in TB1 values, whereas 55.6% (10/18) showed variability in TB2, suggesting higher variability in the TB2 response (Table 6). Nonetheless, no significant differences were identified between pre- and post-treatment values for either TB1 or TB2. Furthermore, the majority of individuals exhibited consistent results over time, indicating that fluctuations were not associated with treatment status. These findings are consistent with prior studies suggesting that IGRAs, as immune response assays, are subject to modulation by host-related factors, such as sex, age, and immune status, thereby limiting their reliability as biomarkers for treatment monitoring (21, 25). Studies with larger cohorts and extended follow-up are needed to validate these observations and enhance the interpretive framework for QFT-Plus dynamics in the context of LTBI therapy.

This study has several limitations. First, although follow-up intervals were stratified into three categories, the precise timing of reversion or conversion within each interval could not be determined from the available data. Nevertheless, categorizing follow-up periods represents a methodological strength beyond a simple binary follow-up analysis. Second, the causes of reversion and conversion remain unclear. Although several factors that may influence variability in IGRA results—such as preanalytical, analytical, postanalytical, manufacturing, and immunological factors—have been previously described (25, 26), this study did not investigate these in detail. Third, the number of individuals undergoing follow-up testing was limited. In clinical practice, follow-up testing is performed for diverse reasons, particularly after LTBI treatment. This study exclusively focused on cases with borderline results. Broader data sets, including non-borderline cases, are necessary for further evaluation. Fourth, as the study was conducted in a country with an intermediate tuberculosis burden, the findings may not be generalizable to settings with low or high TB incidence, where IGRA variability over time may differ.

In conclusion, this study represents the first longitudinal analysis of individuals with borderline QFT-Plus results conducted in an intermediate TB burden setting. Consideration of both TB1 and TB2 tube responses may aid interpretation in the borderline range, although particular caution is warranted when both values fall within this zone. With the accumulation of larger data sets, more refined and standardized interpretive criteria can be established. Moreover, further research on follow-up testing in the borderline range—across high, intermediate, and low TB incidence settings—is essential to validate these findings and to enhance both the diagnostic accuracy and clinical utility of QFT-Plus.

## Data Availability

Data from this study will be made available from the corresponding author upon reasonable request.
